# A Temporal Network Model for Livestock Trade Systems

**DOI:** 10.3389/fvets.2021.766547

**Published:** 2021-12-13

**Authors:** Sara Ansari, Jobst Heitzig, Laura Brzoska, Hartmut H. K. Lentz, Jakob Mihatsch, Jörg Fritzemeier, Mohammad R. Moosavi

**Affiliations:** ^1^Department of Computer Science and Engineering, School of Electrical and Computer Engineering, Shiraz University, Shiraz, Iran; ^2^Department of Complexity Science, Potsdam Institute for Climate Impact Research, Potsdam, Germany; ^3^Institute of Epidemiology, Friedrich-Loeffler-Institut, Greifswald-Insel Riems, Germany; ^4^Landkreis Osnabrück, Veterinärdienst für Stadt und Landkreis Osnabrück, Osnabruck, Germany

**Keywords:** livestock trade, complex network, epidemiology, node centrality measure, temporal network

## Abstract

The movements of animals between farms and other livestock holdings for trading activities form a complex livestock trade network. These movements play an important role in the spread of infectious diseases among premises. For studying the disease spreading among animal holdings, it is of great importance to understand the structure and dynamics of the trade system. In this paper, we propose a temporal network model for animal trade systems. Furthermore, a novel measure of node centrality important for disease spreading is introduced. The experimental results show that the model can reasonably well describe these spreading-related properties of the network and it can generate crucial data for research in the field of the livestock trade system.

## 1. Introduction

In view of recurring large-scale disease outbreaks among different animal holdings, such as that of foot and mouth disease in the UK in 2001 (1), the swine fever epidemics in the Netherlands and Germany in the 1990s and in 2003 (2) and the recent African swine fever (ASF) outbreak in Eastern Europe (3) and China in 2018 and 2019 (4), studies of the systemic reasons for such spreading dynamics are highly relevant for both health and economic reasons. China's outbreak of ASF in 2019 was more devastating than expected (5). This virus, which is harmless to humans but deadly to pigs, has wiped out more than one-third of China's hog population and sent pork prices skyrocketing.

The crucial role of translocation of both domestic and wildlife animals in the emergence of new diseases as a great threat for not only animals but also human health was already identified and discussed in the literature (6–14). These movements are extremely risky that can lead to the introduction of exotic animal diseases or zoonotic human pathogens like monkeypox in the United States in 2003, which infected humans as well (11). Furthermore, trade restrictions cause enormous financial losses for the affected livestock holdings and countries (2, 11). All these can lead to irreparable impacts on the economy and public health (2, 5, 15–17).

Detection of an outbreak as accurately and early as possible when it has not yet been widely spread is thus very important. This gives enough time to authorities for disease management and implementing control strategies on a smaller scale of the population, which is not only easier but also more cost efficient. Data sources such as the one used in this paper (18) are required for risk assessments of epidemics like ASF. Unfortunately in most cases, researchers do not have access to the data crucial for their research because the data are confidential or otherwise unavailable (14). To our knowledge, no synthetic trade data are available for use in numerical analyses and experiments as a substitute for real data or to complement real data.

In this paper, we provide an alternative way to obtain sufficient amounts of data suitable for modeling and studying disease outbreaks in animal trade networks. Our approach is based on large ensembles of *synthetically generated yet realistic* animal trade data. For the production of such data, we design and use a *random animal trade transmission network model* that represents in a stylized fashion the major processes that determine the times, sources, destinations, and sizes of animal transmissions, which occur in a typical animal trade network. In contrast to other more complex agent-based models (19, 20), our model manages to produce realistic distributions of spreading-relevant network features without having to make detailed assumptions about geographic or economic aspects of the trade.

Our model only uses heterogeneous farm capacities and transmission batch sizes. Still, it produces realistic heavy-tailed distributions of certain indicators of a node's importance for disease spreading and detection. As we will see, these distributions are very similar to those observed in real data. For comparison, we fit our generic model to the parameters of the German pig trade network.

## 2. Materials and Methods

### 2.1. Dataset

According to the EU directive EC/2000/15, EU member states collect and record livestock movement data in a national database (12).

According to the German Animal Movement Directive (Viehverkehrsverordnung), each holding is obligated to record every livestock movement within 14 days (18). *HI-Tier* is a comprehensive database that provides the daily description of pig movements in Germany since 2006 (18). In this paper, we use an extract of this database, which contains all trades between premises in Germany between January 1, 2011 and December 31, 2014. The trade data include a set of transitions that comprise information about the seller premise's identifier as the source of movement, the buyer premise's identifier as the target of movement, the number of animals (the *batch size*) and the date of movement. Our dataset contains more than 6.3 million such transactions between 97, 971 premises. After looking closer into data, we noticed that about 40,381 farms did less than 10 trades during 4 years observation period, so we decided to leave out these farms from the rest of analysis. Therefore, the total number of farms reduced to 57,590.

### 2.2. Network Representation

The considered pig trade system can be represented as a temporal network G=(V,E), consisting of a set *V* of premises as the nodes of the network and a set of movements/trades between farms as edges E, where every edge (*i, j, t, w*) is a temporal weighted link representing a trade between a seller and a buyer farm. Therefore, the pig trade network is a large weighted directed *temporal network*. By aggregating all pig displacements over time, also a *static network* view of the data can be obtained, named as *G* = (*V, E*).

**Figure 1:**
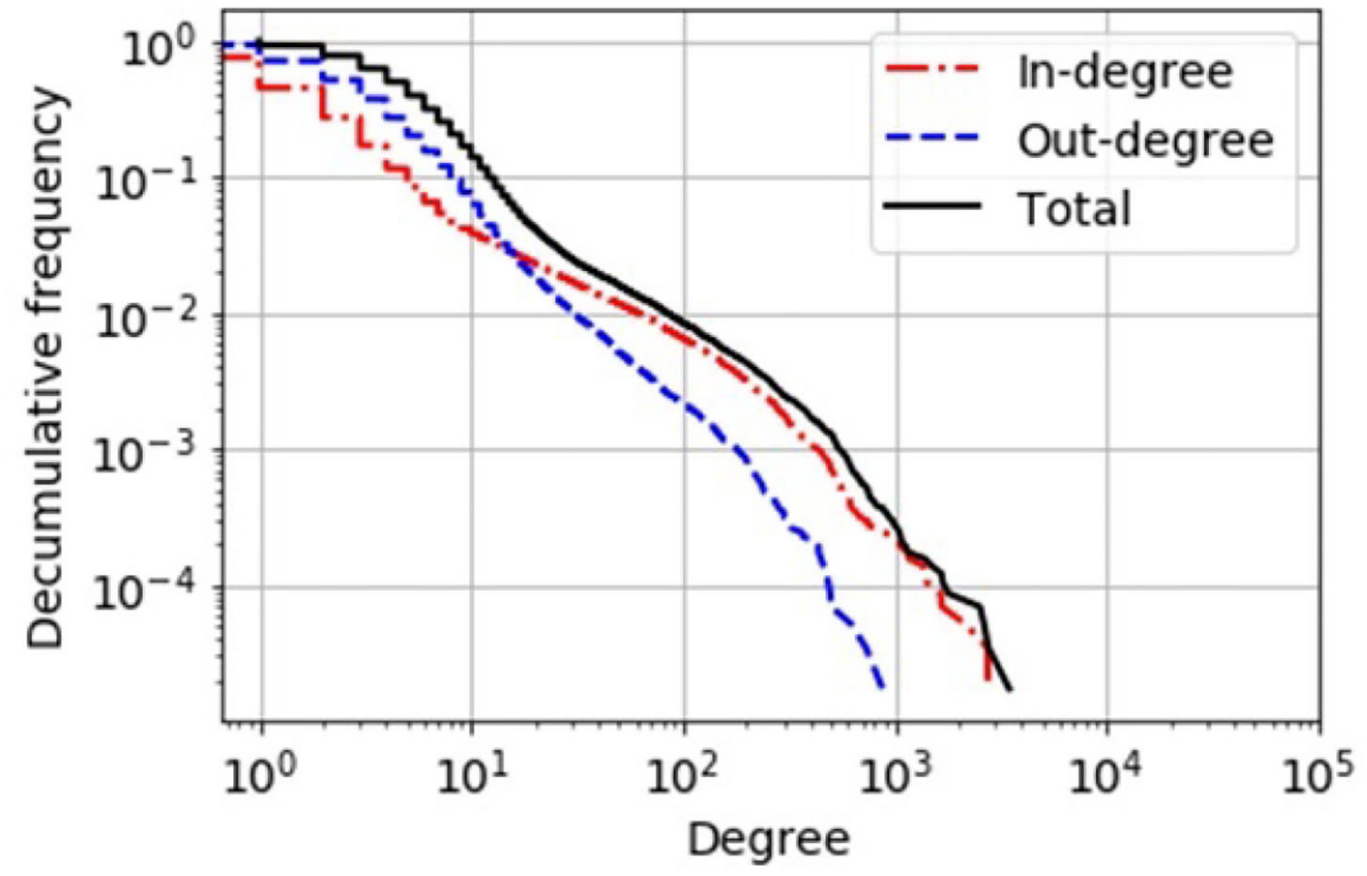
Decumulative degree distribution of the static network view of the German pig trade. In-degree, number of different suppliers of a holding; out-degree, number of different customers of a holding. By comparison, a simple “scale-free” network topology would have a power-law-shaped distribution that would appear as a linear curve in this double-logarithmic plot.

**Figure 2:**
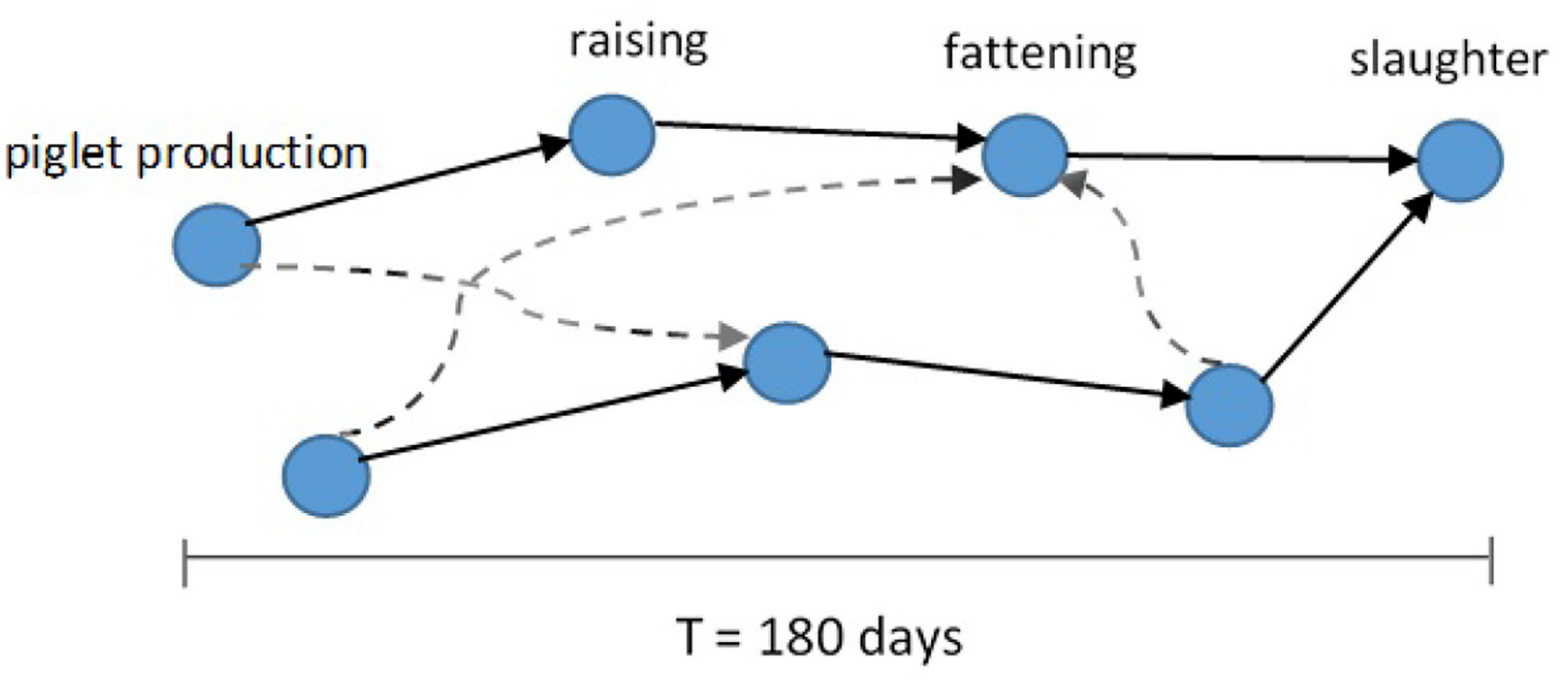
Schematic of pork production chains (solid arrows) forming the pig trade network. Different production chains can be connected by additional cross links (dashed arrows) (13).

We summarize some properties of these two network representations in Figure 1 and Table 1. One special characteristic of the pig trade network is that it is highly determined by the underlying pork production chain which is shown schematically in Figure 2. We conceptualize the chain as consisting of four farm types whereby every farm type is specialized in one of the following steps: piglet production, raising, fattening, and slaughterhouse. As shown in Koeppel et al. (21), the different stages within the production can be determined via the weight limits of the pigs, which are specific to each production step. In addition to the types shown in the figure, we consider the additional node type of “trader” since they are often involved in the trading process and may play an important role in the spreading of an infection due to their high connectedness in the network (see section the set of transmission types). Although in reality there are also some “mixed”-type holdings which do two or three of the above activities, these are rare enough so that we chose to disregard them in our analysis for the sake of conceptual clarity. It is to note that, however, farms are often involved in different individual chains by buying from and selling to more than one partner (dashed lines in Figure 2).

**Table 1:** Basic statistical features of the pig trade network, including the number of nodes *n*, the number of edges *e*, and the average degree 〈*k*〉.

**Type of network representation**	**n**	**e**	**〈*k*〉**
Static network	57,590	258,333	4.486
Temporal network	57,590	6,241,634	108.381

### 2.3. A Novel Node Centrality Measure Related to Disease Spreading

In recent years, numerous measures have been proposed to quantify how important a node in a network is for different aspects. Most of the existing node measures such as degree, closeness centrality (22), betweenness (23), or *k*-shell centrality (24), take only topological, non-dynamical features of the static network view into account for locating the influential nodes. This is why they are not particularly well designed to be informative about dynamic processes occurring on a temporal network such as disease spreading (25).

Some studies that aim at finding influential spreaders in disease spreading dynamics (25–28) define and use tailormade importance measures. One of these is the so-called *dynamic-sensitive* centrality measure that integrates topological properties of the network and spreading dynamics to locate influential spreaders (25). It considers a simple discrete-time spreading process on a static network and defines a spreading influence measure for every node. The spreading influence of node *i* at time step *t* is quantified by the sum of the infection probabilities of all nodes if *i* is the initially infected seed node.


(1) $$\begin{array}{l} S_{i}\left(t\right)={\left[{\left(\beta A+\beta AH+...+\beta AH^{t-1}\right)}^{⊤}L\right]}_{i}. \end{array}$$

where *A* is the adjacency matrix of the network, β is the spreading rate, *H* = β*A* + (1 − μ)*I*, *I* is the identity matrix, μ is the recovery rate, and *L* = [1, 1, ..., 1]^⊤^.

There is also an extension of this approach for temporal networks, the so-called *temporal dynamic-sensitive centrality measure* (28).

By locating the influential spreaders in a trade network, authorities can target them to implement surveillance strategies (29–31). Using Italian cattle displacement data, Bajardi et al. (29) proposed a method for identifying promising nodes where one could place *sentinels* (permanent observation points) by seeking farms, which are more likely to be infected during the early phase of an outbreak. They first find all invasion paths in the network and then cluster all farms with respect to the similarity of their invasion paths. Finally, promising sentinels are determined by selecting nodes from the found clusters. The authors of Schirdewahn et al. (32) showed that this approach also works for the German pig trade network. In this paper, we add another methodology by defining a novel temporal centrality-type measure that reflects a node's importance for achieving the main goal of authorities assumed here, which is to detect an outbreak in as “early” a stage as possible in terms of its impact. This means that, when the outbreak is detected, the number of the infected animals should be as small as possible. Our importance measure captures this goal in a worst-case fashion in order to be independent of any particular infectious disease's specifics. In this way, the measure only depends on the network topology and can be interpreted as an inherent property of the network. We define the *spreading detection turnout centrality* of *j*, denoted *SDTC*(*j*), as the inverse of the expected value of the total weight of all infected nodes when an infection starting at a randomly chosen node *i*_0_ and time *t*_0_ reaches node *j*:


(2) $$\begin{array}{l} SDTC\left(j\right)=\frac{1}{{\langle W\left(i_{0},j,t_{0}\right)\rangle }_{i_{0}\in V\backslash \left\{j\right\},t_{0}\in T}}. \end{array}$$

where *V* is the set of nodes and *T* is set of time points covered by the data. Here, *W*(*i*_0_, *j, t*_0_) is the total weight of all nodes that are infected at the time point at which the outbreak reaches node *j*, assuming that the outbreak starts at a random node and time and that every contact transmits the infection with certainty. In our case, the node weight is the number of animals typically residing at the holding since we take the worst-case assumption that in the case of highly infectious disease all animals in a holding might be infected soon after the infection reaches it. The reciprocal of this measure, 1/*SDTC*(*j*), can thus be interpreted as the average “turnout” of an outbreak of a highly infectious disease if one stops any further spreading as soon as the outbreak reaches node *j*. For any real-world outbreak, the actual number of affected animals would then be at most as large as this number.

The good candidates for surveillance goals are those nodes that typically get infected very early when the outbreak starts at some random position in the network, and they are those with high values of *SDTC*. Hence, the larger *SDTC*(*j*) [i.e., the smaller 1/*SDTC*(*j*)], the more important the surveying of *j* appears for keeping outbreaks small.

### 2.4. The Temporal Network Model

The main contribution of this paper, in addition to the above-discussed importance measure, is an algorithm (a “model”) for generating synthetic but realistic temporal networks representing animal trade, which we present now.

To this end, we represent an evolving animal trade network by a formal data structure of what we call a transmission network. A *transmission network* is simply a finite set *G* of *transmissions* (*t, i, j, x*). Each such tuple (*t, i, j, x*) represents a transmission of *x* many animals from facility *i* to facility *j* at time *t*. In this section, we first describe the basic logic of our model and then discuss its parameters and give the actual algorithm.

#### 2.4.1. Basic Logic of the Model

Our *random animal trade transmission network model* considers a randomly drawn set *N* of hypothetical farms and traders with different characteristics and then generates a hypothetical sequence of animal transmissions (*t, i, j, x*) that may happen between the nodes within some time interval [0, *T*].

Here, *x* is the number of animals of the considered species transported from node *i* to node *j* at a time point *t*.

Since the main use of the generated data is for the study of epidemic spreading through animal trade, each node represents an epidemiological unit (e.g., a barn) wherein livestock may be located at some point in time and which is contiguous enough so that the disease under consideration can potentially spread within the same unit without the need of explicitly transporting animals between different locations. For simplicity, we assume these nodes are either individual *barns* or animal *traders*. Each barn node houses animals of a particular *stage*, *s*, in the livestock's production chain. Also, each trader node specializes in the trade of animals coming from a particular stage. The different stages of the production chain and trader together form the *node types*, θ, of the model. Even though we focus on the pig trade in this paper, which has a rather linear production chain, the model is designed to be also applicable to other livestock (e.g., cattle) that have more complex production chain that may branch according to different use types (e.g., meat production and milk production) or business models, e.g., organic vs. conventional production. Each animal in a node *i* of type θ is bookmarked to be transferred to a certain target node of some type θ′. All animals in *i* bookmarked for the same target type θ′ form what we call a virtual *queue*, *Q*(*i*, θ′). The type of this queue is denoted as (θ, θ′). Queues have non-varying but heterogeneous *capacities* for holding animals. Animals stay in each queue for at least a pre-specified number of days depending on the type of queue (θ, θ′). Afterward, they are considered “ripe” for the next stage and leave the queue as soon as enough animals are ripe, either being sold or killed. How many are “enough” is determined by a varying *minimum batch size*, *s*(*i*, θ′), that is drawn independently for each transmission from a certain probability distribution that again depends on the type of queue. Sales are represented by *transmissions*, (*t, i, j, x*). Each transmission transfers a *batch* consisting of a varying number *x* of animals from a particular queue, *Q*(*i*, θ′), to a particular target node, *j*, of matching type, θ′. As soon as the number of ripe animals reaches *x*(*i*, θ′), and a target node of type θ′ having enough free capacity can be found, all ripe animals in *Q*(*i*, θ′) are sold as one transmission of size *x*.

With a certain probability (called the *loyalty* of the source node), the target node is the same as for the last transmission from this source; otherwise, it is a random node of proper type and capacity. The loyalty of a node is fixed and drawn in the beginning from a certain probability distribution depending on the node type.

Transmissions arrive on the same day and animals are distributed into the target node *j*'s queues in certain fixed proportions.

**Figure 3:**
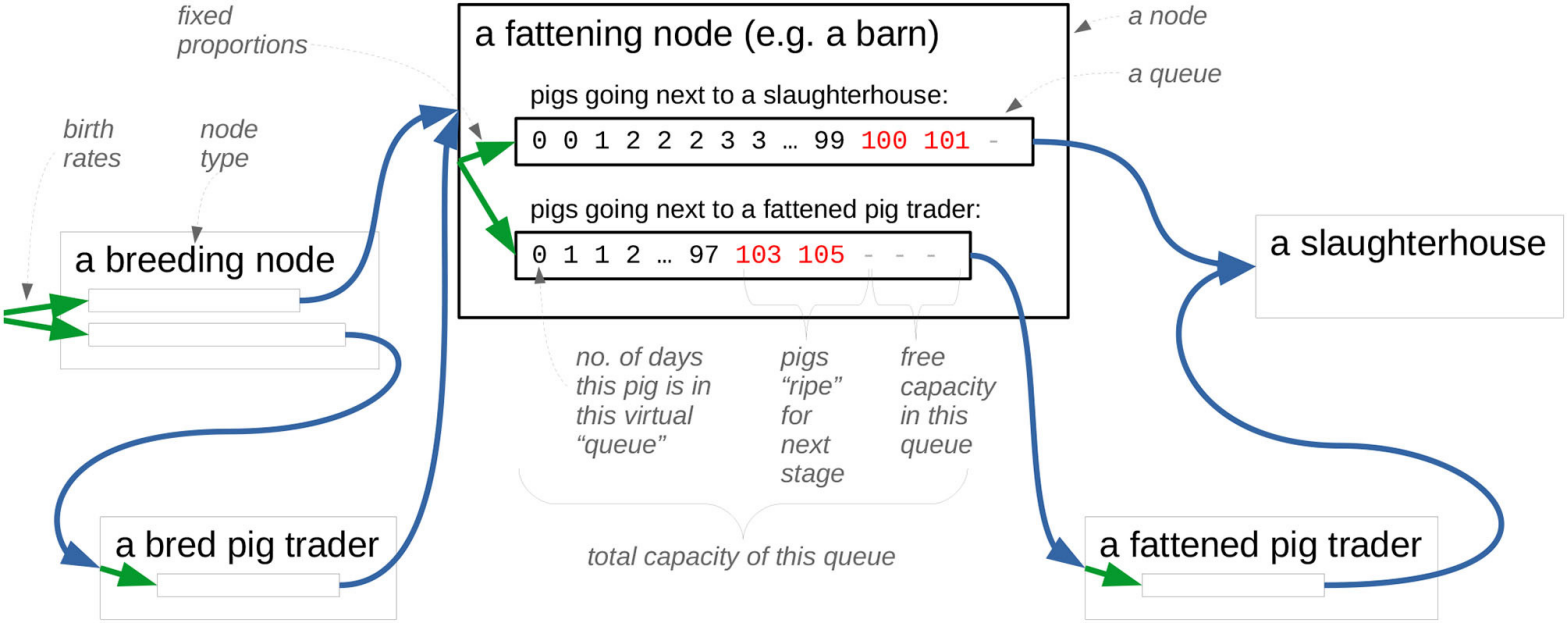
Logic of the random animal trade transmission network model, here for the simple model of German pig trade used in this paper.

Finally, animals are born at fixed rates into certain stages (normally in nodes of type “breeding”), have some daily mortality rate in each stage, and are killed after a specified time in certain other stages (normally in node of type “slaughterhouse” immediately after arrival). Mortality and birth rates are discussed in sections mortality rate and birth rate. There is no additional mortality during transmissions. Figure 3 sketches this logic for the pig trade case used in this paper.

The formal details and simulation algorithm of the model can be found in the Supplementary Materials.

#### 2.4.2. Parameters

- A set Θ_*N*_ of *node types* θ.
- For each type θ ∈ Θ_*N*_, a number *n*_θ_ > 0 of nodes of type θ. Also, a set *N*_θ_ is defined including all node indices of type θ, so that *n*_θ_ = |*N*_θ_|.
- A set Θ_*T*_ of *transmission types* (θ, θ′) in which $\theta ,\theta^{\prime }\in \Theta_{N}$.

For each transmission type $\left(\theta ,\theta^{\prime }\right)\in \Theta_{T}$:

- The relative share $p_{\theta ,\theta^{\prime }}>0$ of animals in nodes of type θ′ that will be transferred to a node of type θ′ next and are thus put in queues of this type.
- A minimal number of days $d_{\theta ,\theta^{\prime }}>0$ each animal spends in a queue of this type.
- A probability distribution $L_{\theta ,\theta^{\prime }}$ for the individual farms' loyalty values ℓ(*i*) that govern the choice of target node.
- A probability distribution $C_{\theta ,\theta^{\prime }}$ for the capacity of queues of this type, typically defined via a tuple of parameters $\gamma_{\theta ,\theta^{\prime }}$ (e.g., the mean and standard deviation of the logarithm of a log-normally distributed capacity, or the exponent and cutoff of a power-law shaped capacity distribution).
- A probability distribution $S_{\theta ,\theta^{\prime }}$ for the minimal batch size (number of animals) of queues of this type.
- A birth rate $b_{\theta ,\theta^{\prime }}\geq 0$ specifying the average number of animals per day and unit of capacity that are born directly into queues of this type.
- A mortality rate $m_{\theta ,\theta^{\prime }}\geq 0$ specifying the probability that an animal in a queue of this type dies during 1 day.

For each type θ ∈ Θ_*N*_, we require that either $\underset{\theta^{\prime }}{\sum }p_{\theta ,\theta^{\prime }}=1$ (non-slaughterhouse nodes) or $\underset{\theta^{\prime }}{\sum }p_{\theta ,\theta^{\prime }}=0$ and *m*_θ_ = 1 (slaughterhouses).

### 2.5. Model Parameters for German Pig Trade

#### 2.5.1. Farm Type

One important value that was not provided in the database and should be inferred is the farm type. To achieve this, we use a simple classification method based on the trade balance ratio and trader index measures, which introduced in Koeppel et al. (21). They considered four classes of farms: breeding, fattening, trader, and slaughterhouse. The *trade balance ratio* **B** gives information about the location and type of a farm in the pork production chain. The balance is the net purchases of a farm normalized by the total trading volume:


(3) $$\begin{array}{l} \text{B}=\frac{P-S}{P+S}. \end{array}$$

where *P* is the total number of purchases of a holding and *S* is the total number of sales of the same holding. Through this definition, the trade balance ratio **B** may take a value between −1 and +1, where **B** = −1 indicates that the farm only sold pigs (e.g., breeding farms), and **B** = 1 means that the farm solely purchased animals (slaughterhouses). If **B** = 0, the purchase volume and sales are equal (traders, fattening farms). To differentiate between fattening and trader farms, they used trader index. Thus, the set of farm types Θ_*N*_ of the pig trade network is:


(4) $$\begin{array}{l} \Theta_{N}=\left\{\text{breeding},\text{fattening},\text{trader},\text{slaughterhouse}\right\}. \end{array}$$

Through this classification, we observe that around 46% of the farms are breeding farms, 35% fattening farms, 0.7% are traders, and 17.5% are slaughterhouses (Table 2).

**Table 2:** Numbers of holding types in the pig trade network.

**Holding type**	**Absolute number**	**Relative number (%)**
Breeding farms	22,536	39.1
Fattening farms	30,879	53.6
Traders	547	0.95
Slaughterhouses	3,718	6.35

It should be noted that this classification is only an assessment. The observed dataset contains only trade information inside Germany and trade contacts between a German farm and a farm abroad are missing. Due to not included contacts, farms which only trade with farms of a foreign country can be considered as slaughterhouses. That is a possible reason for having a high number of slaughterhouses. In Moslonka-Lefebvre et al. (33), another method for the categorization of holdings based on position along the supply chain and degree of market share was proposed.

#### 2.5.2. Farm Capacity

In the case of a disease outbreak, the number of living animals inside a farm affects the total number of infections. Therefore, the farm capacity is a very important factor in the outbreak detection and also in system modeling. On that account, we compute the number of pigs of each premises as the farm's “capacity.” Since these data were not directly available, we estimate the shape of the distribution from the cumulative trading balance in the available data roughly similar to Koeppel et al. (21).

Let *N*(*t*_1_, *t*_2_) be the trading balance (incoming minus outgoing pigs) of some farm between days *t*_1_ and *t*_2_. If the lifestock *S*(*t*) in the farm is assumed to never decrease below a share α of the farm capacity *C* and to have a birth rate of β piglets per capacity and day and a death rate of δ per day, then we know that:


(5) $$\begin{array}{l} C\geq \frac{N\left(t_{1},t_{2}\right)}{1-\alpha -\left(t_{2}-t_{1}\right)\left(\beta -\delta \right)}\;\text{if }1-\alpha -\left(t_{2}-t_{1}\right)\left(\beta -\delta \right)>0, \end{array}$$

and that


(6) $$\begin{array}{l} C\geq \frac{-N\left(t_{1},t_{2}\right)}{1-\alpha -\left(t_{2}-t_{1}\right)\left(\delta \alpha -\beta \right)}\;\text{if }1-\alpha -\left(t_{2}-t_{1}\right)\left(\delta \alpha -\beta \right)>0. \end{array}$$

**Figure 4:**
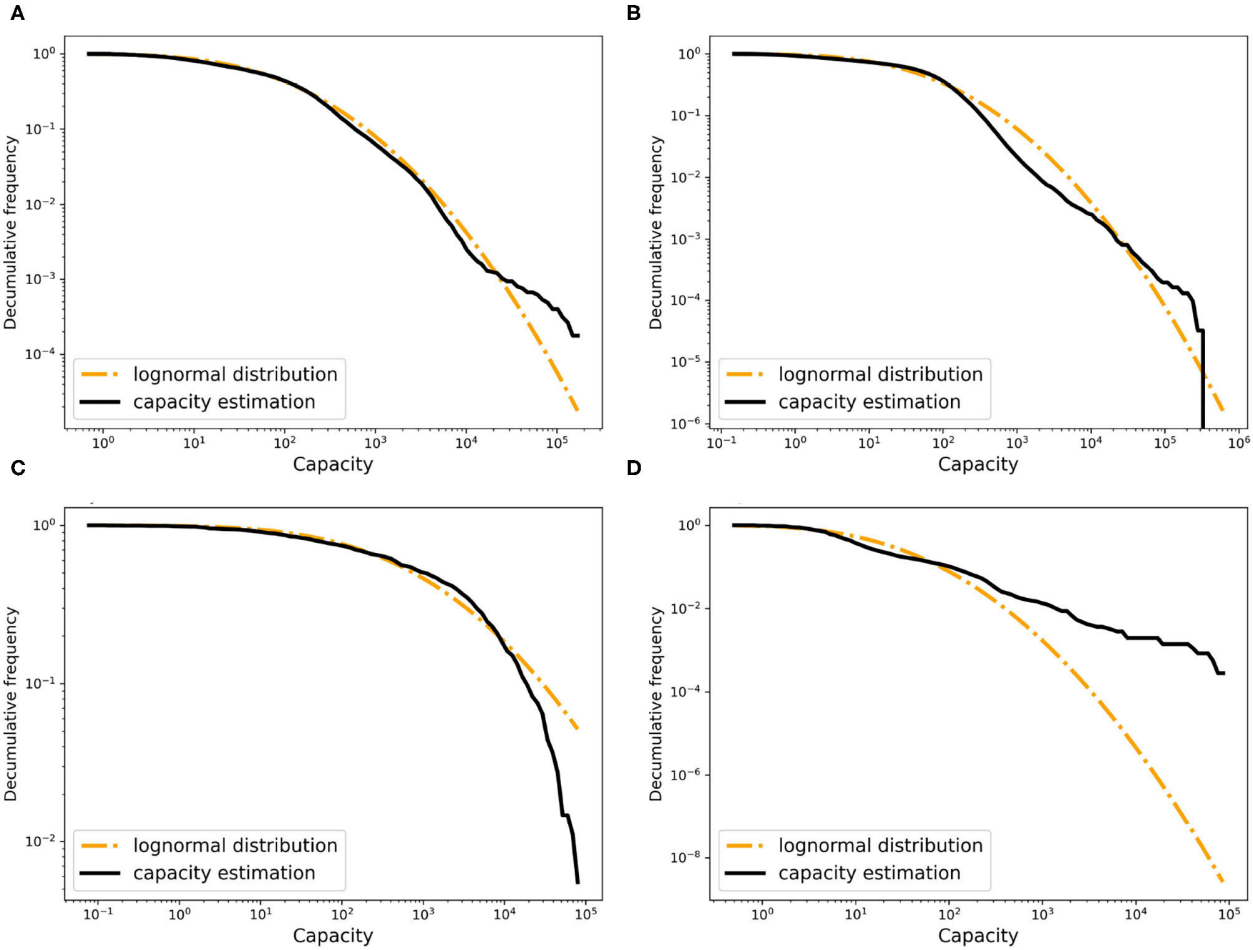
The decumulative distribution of the estimated farm capacity and the fitted random distribution for **(A)** breeding farms, **(B)** fattening farms, **(C)** traders, and **(D)** slaughters.

Computing these for all pairs of time points (*t*_1_, *t*_2_) gives a solid lower bound for the capacity of each farm. We use α = 0.9 and β > 0 for breeding farms and α = β = 0 for other farm types, and use the birth and death rate estimates reported further down. From the resulting bounds, we estimated the shape of the capacity distribution for each farm type. As the capacities of the breeding, fattening, and trader farms were overestimated, we scaled their capacity linearly with a common factor to match the total capacity of 25 million of all farms in Germany. The resulting capacity distributions are shown in Figure 4. As can be seen in the figure, these empirical distributions can be fitted well by lognormal distributions.

#### 2.5.3. The Set of Transmission Types

To determine which transmissions between farm types are more reasonable and should be considered in the model, we study the different movements in the real pig trade network. Regarding the pig production chain, the valid set of movements are those from breeding farms to fattening farms and from fattening farms to slaughterhouses. We consider the two primary pig growing steps as the breeding phase. However, as mentioned before most of the movements are performed via traders in the real system and this case also has been emphasized by different practitioners. Therefore, finding the place of traders in the production chain is important. So for every trade, we analyze the type of the source farm, the destination type, frequency, and the batch size. We consider four classes of farms (breeding, fattening, trader, and slaughterhouses) and analyze all trades between them.

**Figure 5:**
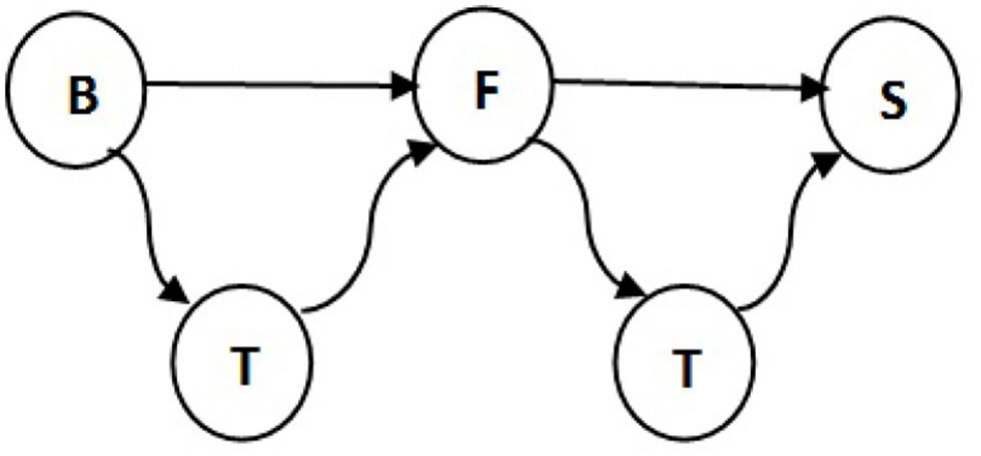
Transmissions between different farm types in the pig trade network. B, F, T, and S indicate breeding farm, fattening farm, trader, and slaughterhouse, respectively.

Table 3 presents the number of pigs traded between every two types of farms. The results support the idea that traders are involved in most of the movements. Note that 57% of total pigs in the breeding stage are moved by traders and only 37% sold directly to fattening farms. We disregard some impossible transitions, for example, this table shows 6% of pigs traded between breeding farms and slaughterhouses and in reality piglets which are 1.5–3 months old need to get weight before being slaughtered. Also traders make 92% of deals between fattening farms and slaughterhouses, therefore the traders are added to the pork production chain as illustrated in Figure 5. All in all, we get our set of transmissions as:


(7) $$\begin{array}{l} \Theta_{T}=\left\{B\to F,B\to T,T\to F,F\to T,T\to S,F\to S\right\}. \end{array}$$

**Table 3:** Number of pigs traded between every two classes of farms.

**Farm type**	**Breeding**	**Fattening**	**Trader**	**Slaughterhouse (%)**
Breeding	0	37%	57%	6
Fattening	2%	0	92%	6
Trader	1%	91%	0	8

*Values are normalized by the total number of pigs sold by all source farms of the same type. We disregard some impossible transitions in the model*.

where Θ_*T*_ contains the transmission types, θ → θ′ is equal to (θ, θ′ ). Here, *B*, *F*, *T*, and *S* are breeding, fattening, trader, and slaughterhouse types, respectively.

#### 2.5.4. Mortality Rate

The mortality rate is the probability of a pig dying within 1 day. This rate depends on the growth stage in pig life. Studies show that most pig deaths happen at the earlier stage of pig's life while few deaths observed in the later growth stages.

For estimation of the mortality rate with use of trade data, the following approach is used. We consider *m*_θ_ as the daily mortality rate in the growth stage θ. Then for each growing phase θ, the *surviving probability* *R*_θ_ can be computed as $R_{\theta }={\left(1-m_{\theta }\right)}^{d_{\theta }}$, which *d*_θ_ is the approximate number of days a pig stays in stage θ. The surviving probability should be approximately equal to the ratio of the total number of sold pigs *S*_θ_ over purchased pigs *P*_θ_ by a farm (fraction of pigs which survived in this stage), *R*_θ_ ≈ *S*_θ_/*P*_θ_(neglecting any growth trend in production). So we can receive the mortality rate *m*_θ_,


(8) $$\begin{array}{l} m_{\theta }\approx 1-{\left(S_{\theta }/P_{\theta }\right)}^{1/d_{\theta }}. \end{array}$$

As an example using pig trade data, we computed the mortality rate in the approximately 100 days long fattening period *m*_*F*_ as:


(9) $$\begin{array}{l} m_{F}\approx 1-{\left(S_{F}/P_{F}\right)}^{1/100}\\ \;=1-{\left(183,772,187/181,225,163\right)}^{1/100}\approx 0.0002. \end{array}$$

This way we could estimate the mortality rate for fattening and trader farms. In slaughterhouses, all pigs are killed, therefore the mortality rate is set to 1 in the model. We disregard death while animals are moving. The pig trade starts exactly after the breeding stage, that is why it is not possible to estimate the animal death rate during breeding stage from equation 10. So further information was required for computing *m*_*B*_. We asked some practitioners to provide us with the real average mortality rate in each pig growing stage. They reported an overall mortality rate of 15–16% in the suckling phase in the first 4 weeks of pig's life, 2–3% in the flatdeck phase in the following 6–8 weeks and the death rate of 2–4% in the fattening stage, which takes 12–14 weeks. These statistics are summarized in Table 4. For estimation of the daily mortality rate using this statistic, we use the average of above values. Thus, the survival rate during the approximately 80 days long breeding stage (including suckling and flat deck phases), *s*_*B*_ = (1 − 15.5%) · (1 − 2.5%) ≈ 0.83 which must approximately equal ${\left(1-m_{B}\right)}^{80}$, hence $m_{B}\approx 1-{\left(0.83\right)}^{1/80}\approx 0.002$. Similarly, $m_{F}\approx 1-{\left(1-0.03\right)}^{1/100}\approx 0.0003$. Therefore, we apply the approximated daily mortality rate 0.002 in the breeding stage in our model, which was computed above based on the practitioner's information.

**Table 4:** Properties of pig growing phase, each phase takes *l* weeks. *m* shows the approximate mortality rate reported by practitioners.

**Pig growing phase**	**l**	**m (%)**
Suckling	4	15–16
Flatdeck	6–8	2–3
Fattening	12–14	2–4

#### 2.5.5. Birth Rate

We define the birth rate *b*_θ_ of the temporal model as the average number of pigs born per day and per unit of capacity in the growth stage θ. It is estimated by the following equation:


(10) $$\begin{array}{l} b_{\theta }=\frac{S_{\theta }}{C_{\theta }d_{\theta }\cdot R_{\theta }}. \end{array}$$

where *S*_θ_ is the total number of pigs sold by farms of type θ and *R*_θ_ is the surviving probability in the stage θ. Hence, *S*_θ_/*R*_θ_ is the approximate total number of pigs born in stage θ, *C*_*B*_ is the total capacity of all farms of type θ and *d*_θ_ as mentioned in previous section is the approximate number of days a pig stays in stage θ. We estimated the daily birth rate 0.087 for the breeding stage and zero for the following stages and apply these values in our model.

#### 2.5.6. The Minimum Size of the Batch

**Figure 6:**
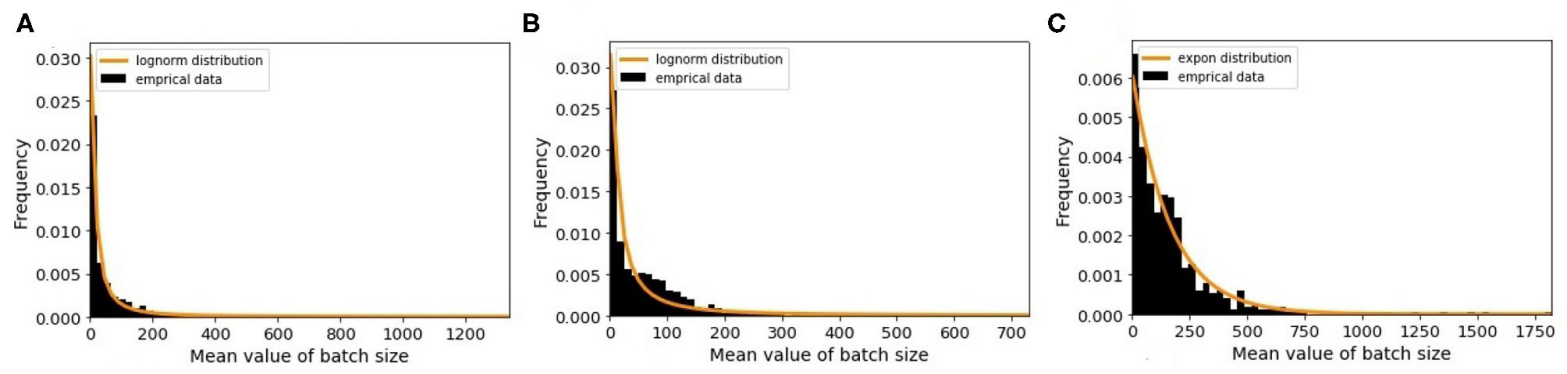
The distribution of mean value of batch size and the fitted random distribution for **(A)** breeding farms, **(B)** fattening farms, and **(C)** traders.

The minimum batch size is defined as the minimum number of animals that farmers prefer to sell to other premises. To estimate this value, we choose randomly 100 farms and plot the distribution of the batch size values. By analyzing the results, we observe that farmers sell different number of animals each time and do not follow a similar pattern. Therefore, we apply a different way for estimation. For this purpose, we randomly select 6,000 farms of each farm type and then plot the distribution of mean value of batch size for every farm type. For traders, we draw only 500 farms as there are only 547 traders. As shown in Figure 6, each farm type has its own batch size distribution. From different probability distribution functions, the best-fitted distributions are log-normal for breeding and fattening types with different parameters and exponential for traders. The R-square values of our fitting are 0.87, 0.80, and 0.93 for above farm types, respectively. We use these probability distributions for generating the minimum batch size value of the queues in the model.

#### 2.5.7. The Loyalty Parameter

The loyalty parameter is defined as the probability of selling animals to the same target as the last time:


(11) $$\begin{array}{l} loyalty\left(i\right)=x_{i}/n_{i}. \end{array}$$

Here, *loyalty*(*i*) is the loyalty value of farm *i* and *x*_*i*_ is the number of times, which farm *i* sells animals to the same destination as the last time. For computing *x*_*i*_ from real data, at first, we set this value to zero for all nodes, then for every outgoing edge of node *i* at time *t*, its destination is compared to the destination of the previous outgoing edge at time *t* − 1.

The value of *x*_*i*_ is increased by one, if they are equal and remains unchanged otherwise. After considering all outgoing edges of *i*, the value of *x*_*i*_ is normalized by *n*_*i*_, which is the total number of outgoing edges of node *i*.

**Figure 7:**
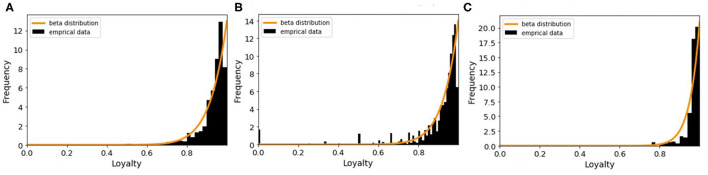
The histogram of loyalty values and its fitted random distribution function for different farm types **(A)** breeding farms, **(B)** fattening farms, and **(C)** traders.

The concept of loyalty also introduced in another study (34) for computing the epidemic risk of each node in the network. Figure 7 illustrates the distributions of loyalty values of the different types. For each farm type, we select the best fitted probability distribution function to the loyalty value. So the beta distribution for breeding, fattening and trader types are applied in the model as $L_{\theta ,\theta^{\prime }}$. The R-square values of our fitting are 0.53, 0.83, and 0.54 for breeding, fattening, and traders, respectively.

## 3. Results

**Figure 8:**
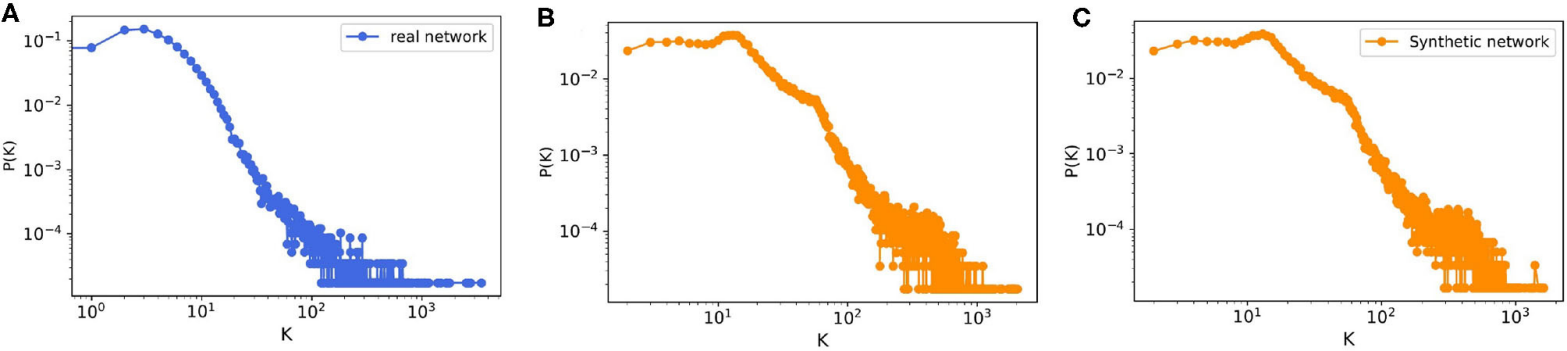
The degree distribution of **(A)** real pig trade network with *n* = 57,590, **(B)** one realization of a synthetic data with *n* = 57,590, and **(C)** ensemble of 10 synthetically generated networks with the same size from the model.

The model output is a dataset with the same format as the real trade data. To evaluate how much our temporal model is capable of reproducing the features of the real-world trade network that are most important for epidemic spreading, we first look at the most basic network characteristic, the in- and out-degree distributions of the real and synthetic networks with 57,590 nodes. Since our model is stochastic, we also compute the degree distributions for a small sample of 10 generated synthetic networks. Figure 8 shows that the resulting degree distributions have a very similar shape as in the real-world network, where most farms have less than 100 trading partners, only few have a large degree, these mostly being farms which have a very large capacity. In addition to the degree distributions, we also compute other relevant network measures such as the size of certain components and the average path length. As in Lentz et al. (13), we consider three different types of components relevant for spreading. First, we looked at the giant strongly connected component (GSCC), which is the largest subset of nodes for which a directed path exists between any pair of them. Then at the giant in-component (GIC), which consists of all nodes outside the GSCC from which there is a path into GSCC. And finally at the giant out-component (GOC), containing all nodes outside the GSCC toward which there is a path from inside the GSCC. As it is shown in Table 5 for the real pig trade network, the GSCC contains 46% of all nodes, and for the synthetic network this fraction is very similar, 43% on average. For the GIC, the match is less accurate but still in the correct order of magnitude, being 34% for the real network and 51% for the synthetic networks on average, hence being too large only by a factor of roughly three halves. Finally, also the GOC size matches reasonably well in terms of order of magnitude, being 10% for the real network and 6% for the synthetic ones on average, hence being too small by a factor of roughly one half.

**Table 5:** Size of components in the real pig trade network and ensemble of 10 generated networks with *n* = 57,590.

**Component**	**Real network (%)**	**Ensemble of generated networks (%)**
*GSCC*	46	43
*GIC*	34	51
*GOC*	10	6
Average path length	5.44	3.88
Number of edges in *GSCC*	116,173	468,194

*GSCC is the giant strongly connected component, GIC is the giant-in component, and GOC shows the giant-out component*.

The average length of undirected paths between any two nodes in the network for the real network is 5.44, compared to 3.88 for the synthetic networks on average.

**Figure 9:**
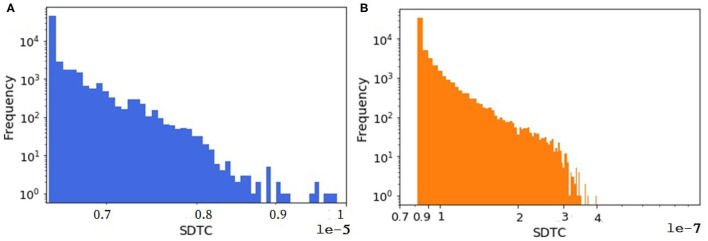
The distribution of weighted *SDTC* for **(A)** the real dataset and **(B)** one realization of a synthetic data. In both networks, *n* = 57,590.

An animal holding with all animals inside is considered as one node in the animal trade network. Since the animal trade network is a weighted network, in another analysis we look at the *SDTC* of the synthetic and the real pig trade network to figure out whether the shape of its distribution is captured by the model. For computing the *SDTC*, the already estimated farm capacity is assumed as the node weight in equation 2 assuming that the economic impacts of an outbreak are strongly related to the total capacity of the affected holdings. We focus on the tail distribution of the *SDTC* measure since the nodes with the largest values of *SDTC* could be used as sentinels for disease surveillance. As shown in Figure 9, the tail distributions of *SDTC* for the real and synthetic networks appear to have the same general shape. The exponential distribution fits well to the tails of *SDTC* for both datasets. The details and related figures can be found in the Supplementary Materials.

These results indicate that the model performs well and the generated data could be used in the different analysis and simulation tasks like disease spreading and a lot of other applications like information dissemination and rumor spreading in the social networks, virus spreading in computer networks, and so on.

## 4. Discussion

Since our goal is early detection of disease, we looked at a disease-oriented temporal centrality measure in section materials and methods. Considering the dynamic characteristics of the animal trade network and disease spreading as a major concern, we designed the *SDTC* measure as a suitable indicator for identifying good sentinels.

However, Figure 9 shows that on average, significantly more animals would be affected by a hypothetical outbreak starting at a randomly selected node in the synthetic network than in the real network, assuming a transmission probability of one. One possible reason for this disparity is that time plays an important role in the computing of temporal measures like SDTC. In our synthetic model, we check all farms daily for animals to be sold. The decision is taken based on the minimum batch size parameter. This means that if the number of animals in the queue would be greater than the minimum batch size, then the farm does sell. Sometimes the minimum batch size (generated from a probability distribution) is small, i.e., one or two. In this case, farms or even traders move a small number of animals immediately, since they always find a slaughter house. Because of this, we have more movements in the synthetically generated dataset (here 1 million more movements) compared to the real one, so we have more paths for spreading in the synthetically generated datasets that lead to more infected cases.

**Figure 10:**
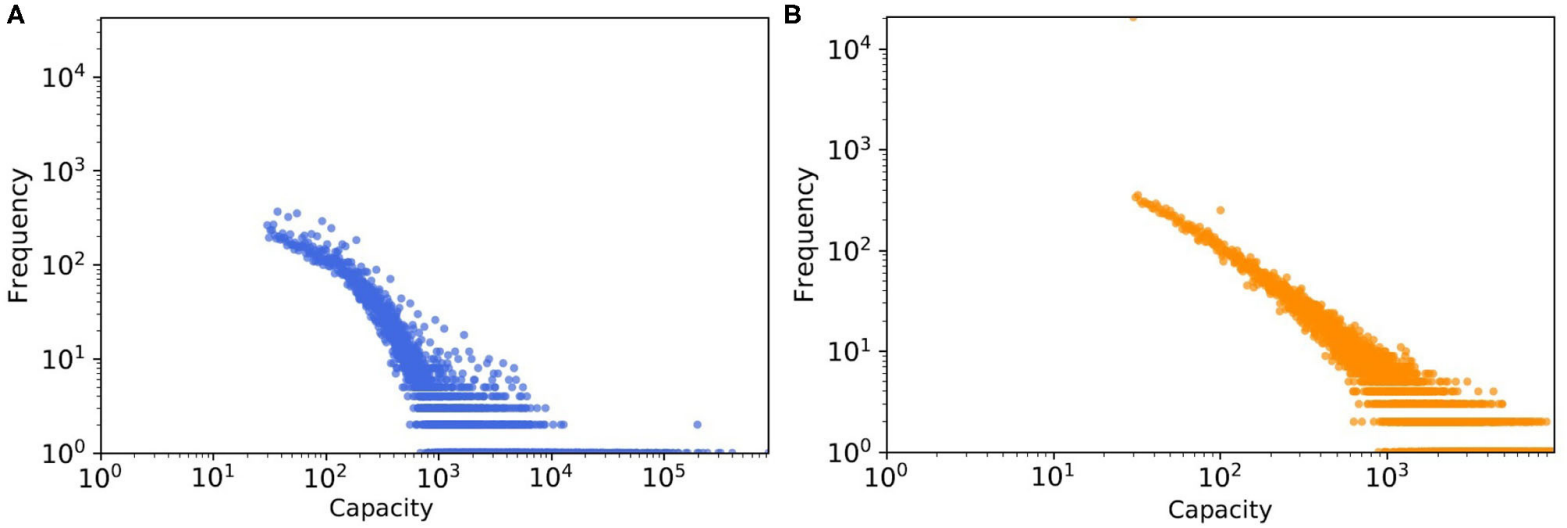
The tail of capacity distribution of **(A)** real pig trade network which estimated from batch size and **(B)** one realization of a synthetic data, here capacity values generated from the fitted probability distribution.

Another factor leading to the disparity in the absolute numbers in Figure 9 might be the quality of the data about farm sizes that was unavailable to us, which we had to estimate quite indirectly from trading volumes (as mentioned in section farm capacity) and then a probability distribution was fitted to this estimated data, not to the real farm sizes (see Figure 10). We expected this mismatch to be in the same order of magnitude for weighted *SDTC* values.

Still, for the selection of sentinels only the relative order of *SDTC* values between nodes matters, not their absolute value, so the shape of the distribution, which matches well between real and synthetic networks, is more important than its location in terms of absolute values. As it is shown in Table 2, about 47% of farms are of breeding type and since they placed at the beginning of the pig production chain, when infection starts from one of the fattening, trader or slaughterhouse farms, they do not get infected any more and therefore have the smallest value of *SDTC* on average. So the breeding farms appear less important nodes for surveillance targets. To the contrary, the important farms with the high values of *SDTC* are in the middle of the pig production chain, such as fattening farms or traders. Since the number of sentinels is small, it is easier and more cost efficient for authorities to implement disease control strategies such as test screening, vaccination, and other countermeasures later on after outbreak detection like trade limitations, isolation, or killing-infected animals in the contaminated holdings.

**Figure 11:**
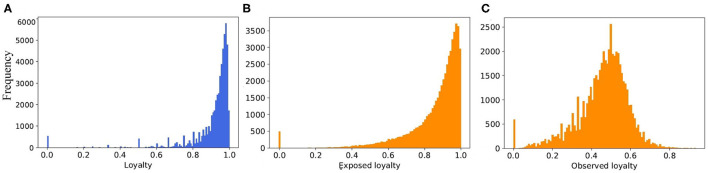
The distribution of loyalty value **(A)** computed from the real dataset, **(B)** generated from the fitted probability function in the model, and **(C)** computed from the output of the model.

When we consider the static view of the real pig trade network, and merge multiple edges between farms, the number of edges from 6.3 million in temporal view reduced to 258,333 in static one. In case of the synthetic networks, however, it is decreased from 7.27 to 1.162 million. Thus, the average degree for static view of real network is around 4.5, and for synthetic it is 20.2. To reduce the average degree of network, we would need to limit the partners of every farm by increasing the loyalty parameter in the model. But this also depends on the capacity of the partners and the generated batch size for every movement. Therefore, if we limited the number of partners to a small value and they have no capacity for buying new animals, the number of total movements (edges) would reduce a lot. This would also have an effect on the structure of network. As it is shown in Figure 11, although the *exposed* loyalty values in the model are very close to the real ones, the *observed* loyalty for the generated network is different. We can state that the degree of the generated networks depends on a combination of loyalty, capacity, and batch size, all of these are taken from different probability distributions. Due to randomness of all these values, some combinations are unexpected, which leads to a decrease in the accuracy of the results.

Another issue regarding the real dataset is missing data. Some farms appear as buyers only and have no selling information. As mentioned in section farm type, in the classification method we could consider them as slaughterhouses and if so, then the number of slaughterhouses in the 4 years observation period increased to 3,627. However, we know that the total number of slaughterhouses in 2011–2015 was roughly 2000. If we would not consider those nodes as slaughterhouses, they could be of any other types like breeding, fattening, or traders. Therefore, the number of farms of each type is not precise and the estimations of all parameters in our model are affected by this issue.

All in all, our simple model was able to reproduce the most important disease-related features of the real network, even though we do not claim that the structure and dynamic of the synthetically generated networks is equivalent to the real network. We believe it is better to have a model that has too many potential spreading paths rather than too few. Hence, we are on the “safe” side by rather overestimating impact than underestimating it, when assessing epidemic impacts.

## 5. Conclusion

Understanding the effects of network structure on disease spreading processes is essential for improving the early detection of outbreaks. To study this, outbreaks on realistic networks must be simulated and analyzed. To generate reliable results, one must make sure that such simulations do not depend on specific peculiarities of the used sample of network data that are not representative of the long-term network structure. Although generalizable insights require the study of many realistic networks, available real-world data are sparse. In this paper, we have shown how to overcome this lack of data by using synthetically generated network data that displays a similar structure to real-world pig trade data. To this end, we presented an algorithm for generating such synthetic data to be used in epidemic simulations. The performance of this model was evaluated by comparing the synthetically generated data with a 4-year interval of the German pig trade network comprising on the order of 100,000 holdings and several millions of animal movements. For this comparison, we used not only standard measures of network-topological features but also a novel measure of node importance for the surveillance of disease spreading called SDTC. Our results show that our algorithm produces data that strongly resemble the real data in terms of these criteria. Epidemic modelers can hence use these synthetic networks to study spreading on realistic networks and derive policy implications. In a follow-up study, we will use the *SDTC* measure to derive better strategies for selecting holdings to regularly test in order to detect outbreaks earlier, and will use our synthetic network data to evaluate these strategies.

## Data Availability Statement

Data on pig movements cannot be made freely available to the general public due to legal restrictions. The reason is that used data contains private information that cannot be disclosed without the individual agreement of each German pig farmer. Data may be provided to any interested party that requests it to the Bayerisches Staatsministerium für Ernährung, Landwirtschaft und Forsten (StMELF). The dataset for this study is available in HI-Tier repository at http://www.hi-tier.de.

## Author Contributions

SA and JH: study conception, design, draft manuscript preparation, developed the theory, and performed the computations. HL and JF: data collection. SA, JH, LB, JM, HL, and MM: analysis and interpretation of results. All authors reviewed the results and approved the final version of the manuscript.

## Funding

This work was supported by the German Bundesministerium Für Bildung und Forschung, FKZ 01KI1812.

## Conflict of Interest

The authors declare that the research was conducted in the absence of any commercial or financial relationships that could be construed as a potential conflict of interest.

## Publisher's Note

All claims expressed in this article are solely those of the authors and do not necessarily represent those of their affiliated organizations, or those of the publisher, the editors and the reviewers. Any product that may be evaluated in this article, or claim that may be made by its manufacturer, is not guaranteed or endorsed by the publisher.
