# Supplementary material for: A Temporal Network Model for Livestock Trade Systems
Source: Front Vet Sci. 2021 Dec 13;8:766547. doi: 10.3389/fvets.2021.766547 (PMC8710670; doi:10.3389/fvets.2021.766547)
Supplement: Supplementary file 1 [file Data_Sheet_1.PDF]

Supplementary Material

1 MODEL ALGORITHM

Initialization.

For each $\theta \in \Theta_N$, let N_θ be a set of n_θ many unique node indices i, representing the barns of this type.

For each transmission type $(\theta, \theta') \in \Theta_T$ and each barn $i \in N_\theta$, let $Q(i, \theta')$ be an empty list, called a 'queue'. At each time point during the algorithm, the queue will contain one entry for each animal currently belonging to the queue, and the entry will specify the number of days the animal already is in this queue. Draw a queue capacity $c(i, \theta')$ from $C_{\theta,\theta'}$ and a loyalty value $\ell(i)$ from $L_{\theta,\theta'}$. Also draw an initial minimal batch size $s(i, \theta')$ from $S_{\theta,\theta'}$.

Finally, draw a 'going destination' $g(i, \theta')$ from $N_{\theta'}$.

Time-forward simulation.

Then, for each day $t = 1, \ldots, T$, all queues are processed in a random order (a new random order of queues for each day). When queue $Q(i, \theta')$ of barn $i \in N_\theta$ is processed:

1. *Aging*. Add 1 to all entries in $Q(i, \theta')$.
2. *Deaths.* Remove each entry in $Q(i, \theta')$ with probability $m_{\theta,\theta'}$.
3. *Births.* Draw a number of births, k, from the geometric distribution with expected value $b_{\theta,\theta'} \times c(i, \theta')$ and append k many zeroes to $Q(i, \theta')$. If necessary, truncate $Q(i, \theta')$ to length $c(i, \theta')$ because of the limited capacity of the queue.
4. *Identify no. of 'ripe' animals.* Let x be the no. of entries in $Q(i, \theta')$ which are $\geq d_{\theta,\theta'}$.
5. *Check transmission necessity.* If $x < s_{\theta,\theta'}$, go to step 11 since the necessary batch size is not yet reached; otherwise proceed as follows:
6. *Preferred target barn j for full transmission.* With probability $\ell(i)$ (loyalty), put $j = g(i, \theta')$, otherwise draw j randomly from $N_{\theta'}$.
7. *Compute free capacity of barn j.* Put $f_j = \sum_{\theta''}[c(j, \theta'') - |Q(j, \theta'')|]$.
8. *Perform transmission and update.* If $f_j \geq x$ (i.e., if j has enough free capacity to receive x animals):
   a) add the transmission (t, i, j, x) to the output data,
   b) update the going destination of this queue to $g(i, \theta') = j$,
   c) remove the first x entries (representing the transmitted animals) from queue $Q(i, \theta')$,
   d) draw a new minimal batch size $s(i, \theta')$ from $S_{\theta,\theta'}$ for the next transmission from this queue, and,
   e) if j is not a slaughterhouse, do the following x times (i.e., once for each animal arriving at j): draw a new target type θ'' according to the probabilities $p_{\theta',\theta''}$ and append a zero to the corresponding queue $Q(j, \theta'')$.
9. *Alternative target for full transmission.* Otherwise (if $f_j < x$), check whether any other potential target barn $j \in N_{\theta'}$ has enough free capacity $f_j \geq x$. If yes, draw one such j at random with probabilities proportional to (full!) capacity $c(j, \theta'')$, and perform step 8 with this j.
10. *Alternative target node for partial transmission.* Otherwise (if no such j exists), check whether $\max_{j \in N_{\theta'}} f_j \geq s_{\theta,\theta'}$, i.e., whether there is at least one potential target whose free capacity exceeds the

current minimum batch size. If yes, draw a j from those potential targets, i.e., from $\arg\max_{j \in N_{\theta'}} f_j$, at random, with probabilities proportional to $c(j, \theta'')$, and perform step 8 with this j and with $x = f_j$. Otherwise (if again no such j exists):

11. *No transmission.* There is no transmission from this queue at this time.

1.1 Model Complexity.

All births, aging, dying, registering operations in the algorithm are of order $O(TNQC)$ and finding the target node for transmission has the complexity $O(TNQN))$. So the time complexity of our proposed model is $O(TNQ(C + N))$ where T is the number of time points (observation period), N is the number of nodes, Q is the average number of queues per node and C is the average capacity of a queue.

2 MORE ANALYSIS OF SDTC MEASURE.

For more analysis of the tail distribution, we ignored the extreme outliers (mostly slaughterhouses) and fitted gamma, exponential, lognormal and power-law distributions to both real and synthetic weighted STDC data. The results show that exponential distribution fitted best to the data (see Figure S1). The R-square value for both real and synthetic data is 0.80. This shows that the model is capable of reproducing the shape of the SDTC distribution reasonably well and this definitely helps in identifying sentinels farms.

3 FIGURES

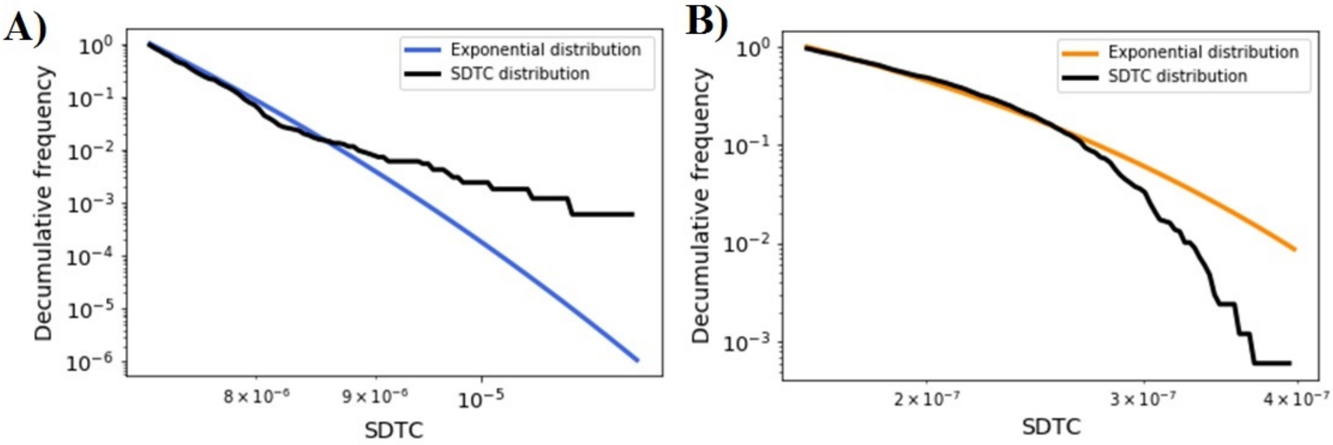

Figure S1. The tail distribution of SDTC and fitted random distribution for A) Real pig trade network, B) One realization of a synthetic data.
