# Supplementary figures and images for: A Temporal Network Model for Livestock Trade Systems
Source: Front Vet Sci. 2021 Dec 13;8:766547. doi: 10.3389/fvets.2021.766547 (PMC8710670; doi:10.3389/fvets.2021.766547)

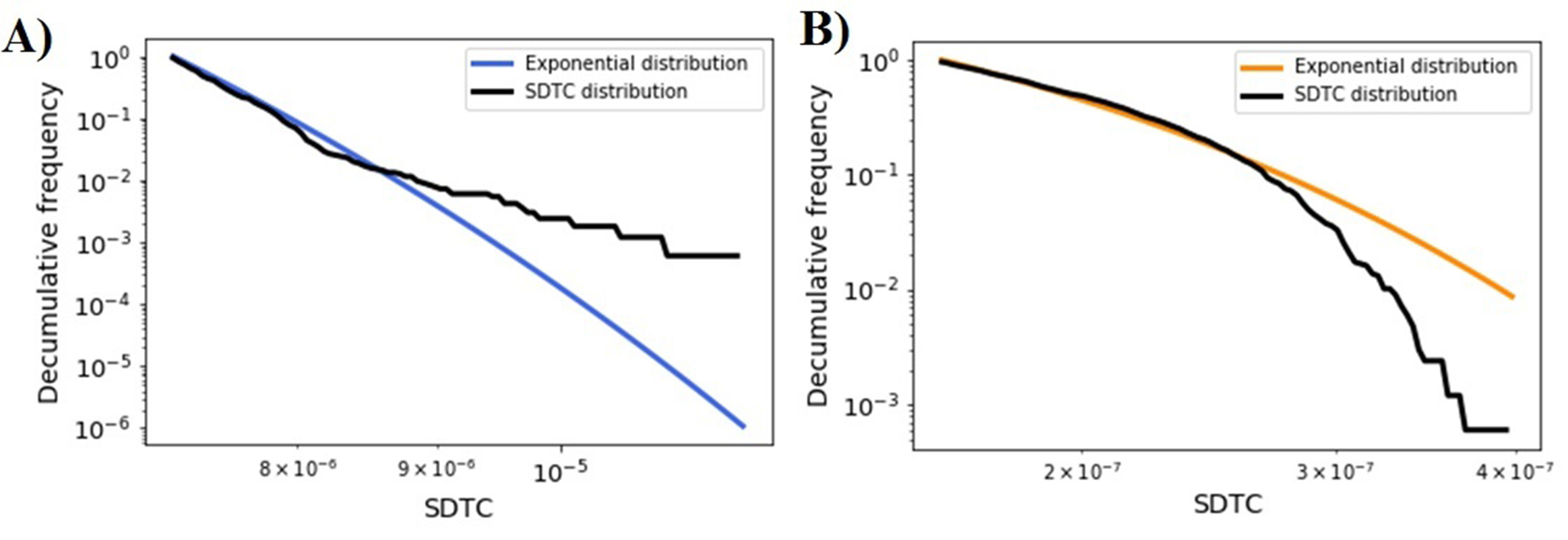

Supplement: Supplementary file 2 [file Image_1.jpg]
